# Benchmarking the ACEnano Toolbox for Characterisation of Nanoparticle Size and Concentration by Interlaboratory Comparisons

**DOI:** 10.3390/molecules26175315

**Published:** 2021-09-01

**Authors:** Ruud Peters, Ingrid Elbers, Anna Undas, Eelco Sijtsma, Sophie Briffa, Pauline Carnell-Morris, Agnieszka Siupa, Tae-Hyun Yoon, Loïc Burr, David Schmid, Jutta Tentschert, Yves Hachenberger, Harald Jungnickel, Andreas Luch, Florian Meier, Jovana Kocic, Jaeseok Kim, Byong Chon Park, Barry Hardy, Colin Johnston, Kerstin Jurkschat, Jörg Radnik, Vasile-Dan Hodoroaba, Iseult Lynch, Eugenia Valsami-Jones

**Affiliations:** 1 Wageningen Food Safety Research, Wageningen University & Research, Akkermaalsbos 2, 6708 WB Wageningen, The Netherlands; ingrid.elbers@wur.nl (I.E.); anna.undas@wur.nl (A.U.); eelco.sijtsma@wur.nl (E.S.); 2 School of Geography, Earth and Environmental Sciences, University of Birmingham, Edgbaston, Birmingham B15 2TT, UK; s.m.briffa@bham.ac.uk (S.B.); i.lynch@bham.ac.uk (I.L.); e.valsamijones@bham.ac.uk (E.V.-J.); 3 Malvern Panalytical, Enigma Business Park, Grovewood Road, Malvern, Worcestershire WR14 1XZ, UK; pauline.carnell-morris@malvern.com (P.C.-M.); agnieszka.siupa@malvern.com (A.S.); 4 Department of Chemistry, College of Natural Sciences, Hanyang University, Seoul 04763, Korea; taeyoon@hanyang.ac.kr; 5 Institute of Next Generation Material Design, Hanyang University, Seoul 04763, Korea; 6 CSEM, Centre Suisse d’Electronique et de Microtechnique SA, Bahnhofstrasse 1, 7302 Lanfquart, Switzerland; loic.burr@csem.ch (L.B.); david.schmid@csem.ch (D.S.); 7 German Federal Institute for Risk Assessment (BfR), Department of Chemical and Product Safety, Max-Dohrn-Strasse 8-10, 10589 Berlin, Germany; jutta.tentschert@bfr.bund.de (J.T.); yves.hachenberger@gmx.de (Y.H.); harald.jungnickel@bfr.bund.de (H.J.); andreas.luch@bfr.bund.de (A.L.); 8 Postnova Analytics GmbH, Rankine-Str. 1, 86899 Landsberg, Germany; florian.meier@postnova.com; 9 Department of Chemistry and Applied Biosciences ETH Zurich, Vladimir-Prelog-Weg 1, 8093 Zurich, Switzerland; jovanat@inorg.chem.ethz.ch; 10 Korea Research Institute of Standards and Science (KRISS), 267 Gajeong-ro, Yuseong-gu, Daejeon 34113, Korea; jaeseok.kim@kriss.re.kr (J.K.); bcpark@kriss.re.kr (B.C.P.); 11 Edelweiss Connect GmbH, Technology Park Basel, Hochbergerstrasse 60C, 4057 Basel, Switzerland; barry.hardy@edelweissconnect.com; 12 Department of Materials, University of Oxford, Begbroke Science Park, Begbroke Hill, Oxford OX5 1PF, UK; colin.johnston@materials.ox.ac.uk (C.J.); kerstin.jurkschat@materials.ox.ac.uk (K.J.); 13 Bundesanstalt für Materialforschung und-prüfung (BAM), Unter den Eichen 87, 12205 Berlin, Germany; joerg.radnik@bam.de (J.R.); dan.hodoroaba@bam.de (V.-D.H.)

**Keywords:** nanomaterials, analysis, benchmarking, interlaboratory comparison

## Abstract

ACEnano is an EU-funded project which aims at developing, optimising and validating methods for the detection and characterisation of nanomaterials (NMs) in increasingly complex matrices to improve confidence in the results and support their use in regulation. Within this project, several interlaboratory comparisons (ILCs) for the determination of particle size and concentration have been organised to benchmark existing analytical methods. In this paper the results of a number of these ILCs for the characterisation of NMs are presented and discussed. The results of the analyses of pristine well-defined particles such as 60 nm Au NMs in a simple aqueous suspension showed that laboratories are well capable of determining the sizes of these particles. The analysis of particles in complex matrices or formulations such as consumer products resulted in larger variations in particle sizes within technologies and clear differences in capability between techniques. Sunscreen lotion sample analysis by laboratories using spICP-MS and TEM/SEM identified and confirmed the TiO_2_ particles as being nanoscale and compliant with the EU definition of an NM for regulatory purposes. In a toothpaste sample orthogonal results by PTA, spICP-MS and TEM/SEM agreed and stated the TiO_2_ particles as not fitting the EU definition of an NM. In general, from the results of these ILCs we conclude that laboratories are well capable of determining particle sizes of NM, even in fairly complex formulations.

## 1. Introduction

An increasing number of nanomaterials (NMs) are entering the market in consumer products spanning from health care and leisure to electronics, cosmetics and foodstuff. Nanotechnology is a truly enabling technology, with unlimited potential for innovation and numerous benefits derived from the NM unique size-related properties that are widely recognised [1,2]. However, the novel and dynamic properties and range of forms of NMs make the development of a well-founded and robust legislative framework to ensure the safe development of nano-enabled products particularly challenging. Legislation requiring the labelling of the presence of NMs in cosmetics [3], food [4] and biocidal products [5] containing NMs exists at EU level, where a recommendation for a definition of NMs has been published [6] specifying 50% of the particles by number to be less than 100 nm. Assessing whether the requirements for labelling are implemented correctly requires effective control, based on reliable measurements that are able to determine number-based particle size distributions. Of the most commonly used techniques for the determination of the diameters of NM, only electron microscopy (EM), particle tracking analysis (PTA), and single-particle inductively coupled plasma mass spectrometry (spICP-MS) deliver number-based particle size distributions. While some of these techniques have been around for decades (e.g., EM), others have been on the market over 10 years (PTA) or are recent extensions of established methods such as ICP-MS to detect particles in addition to total metal concentration. Thus, method development and optimisation for NMs size and concentration determination is a continuous process. Over time, these are refined to implement technical advances in terms of sample introduction and/or detection as well as software advances to enhance particle detection, counting and image analysis including high-throughput computational approaches which are emerging rapidly, driven by both regulatory and research demands. Recently, ISO and CEN documents have become available, describing the use of these techniques and optimized protocols for the size determination of NMs [7,8,9]. However, as can be expected of rapidly evolving techniques, data on interlaboratory comparisons (ILCs) to determine the performance of the methods or laboratories applying these methods are quite sparse. Transmission electron microscopy (TEM) is an exception since it is one of the most widely used methods for the characterisation of NMs and several ILCs have been organised in the past. These include ILCs of gold nanoparticles (NPs) [10], gold nanorods [11], cellulose nanocrystals [12], titania aggregates [13] and carbon black aggregates [14]. spICP-MS was among the methods investigated in the EU-funded project “NanoLyse” which aimed at developing and validating methods for the detection and quantification of nanoparticles (NPs) in food. Within that project, an ILC for the determination of median particle size and particle number concentration of silver NPs in food simulants was organised [15]. At a later point in time a similar interlaboratory method performance study for the size determination and quantification of silver NPs in chicken meat by spICP-MS was organised [16]. Similarly, the EU-funded “QualityNano” project organised a set of ILCs assessing the capability of PTA for the determination of gold NPs in water and medium containing the model protein bovine serum albumin, as well as particle mixtures [17]. Recently, the results of a further ILC using PTA for the size determination of gold NP in aqueous media and that of an ILC of spICP-MS for the size determination of TiO_2_ particles in confectionery products were published [18,19].

PTA, spICP-MS and TEM are, with other analytical techniques, part of the ACEnano toolbox. ACEnano is an EU-funded project which aims at developing, optimising, benchmarking and validating methods for the detection and characterisation of NP with a focus on extending the applicability of those methods that are already quite well established for NM characterisation in simple media to more complex and more realistic matrices including biological and environmentally relevant media and commercially formulated matrices. Within this project, several ILCs for the determination of particle size and concentration have been organised to test and benchmark the performance of routinely available analytical methods. Methods for which ILCs have been organised so far include PTA, spICP-MS and TEM/SEM, as well as dynamic light scattering (DLS), asymmetrical flow field-flow fractionation (AF4), the Brunauer, Emmett and Teller method (BET) for specific surface area and ultraviolet-visible spectrophotometry (UV/VIS). In this publication we report the results of the ILCs for a number of these techniques measuring pure NPs in aqueous suspension as well as in commercially available consumer products including sunscreen lotion and toothpaste.

## 2. Results

### 2.1. Measurements of Pristine Nanoparticles

At first, pristine nanoparticles were used in the ILCs to determine whether methods and laboratories were able to determine accurate particle sizes in simple matrices before going to more complex matrices like consumer products. The techniques that were tested in these ILCs were PTA, spICP-MS, TEM/SEM, DLS and AF4. The results are presented here and the reported results of all participants in the ILCs can be found in the supplemental information.

#### 2.1.1. Particle Tracking Analysis

**Figure 1:**
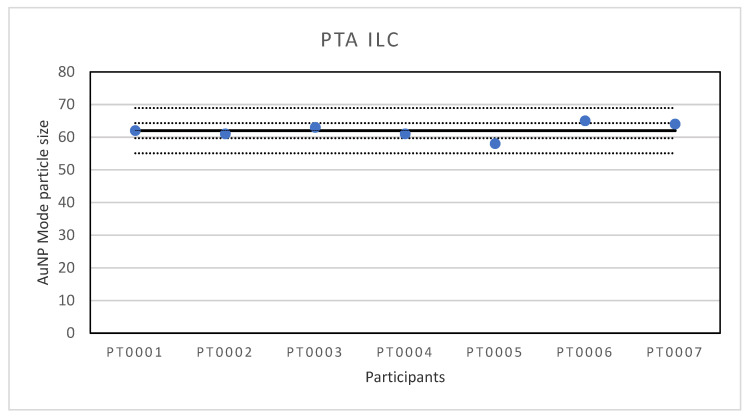
Results for the particle size reported by participants in the PTA ILC. The straight line indicates the consensus value, the dotted lines one and three times the robust standard deviation.

A first ILC was organised for the determination of particle size of Au NPs with PTA. PTA, also called nanoparticle tracking analysis (NTA), is a particle characterization technique which can measure the hydrodynamic size and NPs concentration in liquid suspension. For PTA measurements, NPs in liquid suspension are loaded into a sample chamber, which is illuminated by a specially configured laser beam. Light scattered by particles in the path of the beam as they move under Brownian motion is collected with a digital camera, allowing individual particles to be identified and located within the field of view. Using image analysis, each particle’s position is tracked over subsequent camera frames to calculate the mean square displacement in 2-dimensions and determine the diffusion coefficient. The diffusion coefficient is then used to calculate the hydrodynamic diameter, which is the diameter of the particles including the solvent molecules which move around with the particles [20]. Seven laboratories participated in the ILC, six were situated in Europe and one in Asia. The lowest reported value for particle size is 58 nm and the highest value 65 nm. The results of all participants are shown in Figure 1.

The consensus value is 62 nm with a robust standard deviation of 2.3 nm. This is more than two times lower than the fit-for-purpose value of 10% of the consensus value (=6.2 nm) indicating a good performance. The uncertainty of the consensus value is 1.09 nm which does not exceed 0.3σ_p_ and therefore the uncertainty is not taken into account in the calculation of the z-scores. With regard to the z-score accuracy, all results were satisfactory.

#### 2.1.2. Single Particle ICP-MS

**Figure 2:**
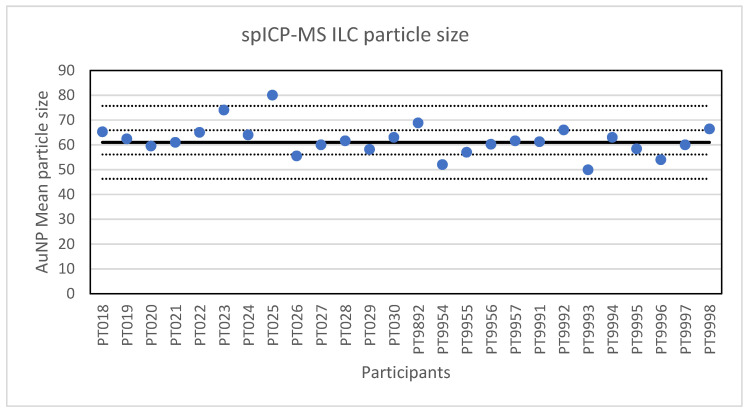
Results for the particle size reported by the participants in the spICP-MS ILC. The straight line indicates the consensus value, the dotted lines one and three times the robust standard deviation.

The second method selected for an ILC was spICP-MS. In spICP-MS, NP in an aqueous sample suspension and with an elemental composition compatible with ICP-MS, are introduced into the ICP-MS producing a plume of element ions in the plasma torch. This plume is detected as a signal pulse in the mass spectrometer and allows the determination of the particle concentration in the suspension as well as the mass of the element in the individually detected NPs. Based on the particle mass, composition, density and an assumed particle shape, the particle size can be estimated. Adequate time resolution (typically in the range of 100 µs to 10 ms) and a low particle concentration (typically in the range of 0.01 to 1 µg/L) are required to ensure that each signal pulse originates from one particle only [21]. For the ILC, a material was prepared by diluting a NanoComposix citrate-stabilised 60 nm gold nanoparticle suspension. Twenty-six laboratories participated in the ILC and each laboratory was asked to report the mean particle diameter and the particle number concentration through the web application designed for this purpose. All laboratories reported results for the particle diameter. The lowest value reported was 49.9 nm and the highest value 80 nm. A presentation of all reported values in given in Figure 2. The consensus value is 61 nm with a robust standard deviation of 4.9 nm. This is lower than the fit-for-purpose value of 10% of the consensus value (=6.1 nm) indicating a good performance. The uncertainty of the consensus value is 1.2 nm which does not exceed 0.3σ_p_ and thus the uncertainty was not taken into account when calculating the z-scores. With regard to the accuracy, one result was questionable and one was unsatisfactory.

**Figure 3:**
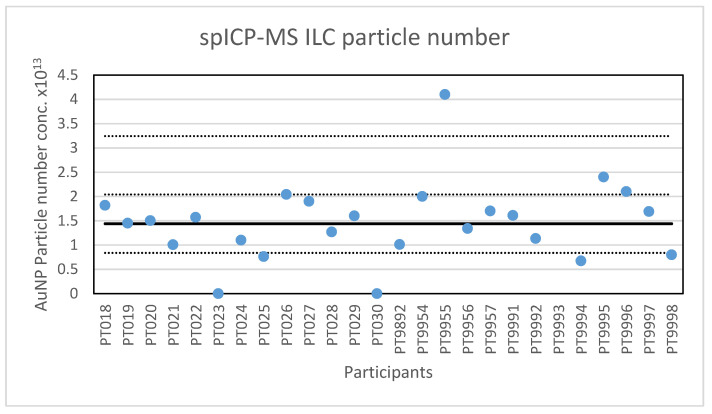
Results for the particle number concentration reported by participants in the spICP-MS ILC. The straight line indicates the consensus value, the dotted lines one and three times the robust standard deviation.

In addition to particle size, participants were asked to report the particle number concentration. All but one of the laboratories reported results for the particle number concentration. After reporting the results, seven participants were asked to check their reported concentration one more time, since it appeared that the dilution factor was not taken into account or that the result was reported per millilitre and not per litre. Four laboratories corrected their results while three results remained deviating. With this, the lowest value reported is 1.73 × 10^7^ parts/L and the highest value is 4.1 × 10^13^ parts/L. The consensus value is 1.44 × 10^13^ parts/L with a robust standard deviation of 0.6 × 10^13^ parts/L. This is more than twice the fit-for-purpose value of 20% of the consensus value (=0.29 × 10^13^ parts/L) indicating that the determination of the particle concentration is far more difficult then determining particle size. The uncertainty of the consensus value is 0.15 × 10^13^ parts/L which exceeds 0.3σ_p_, and thus the uncertainty is taken into account when calculating z-scores. A graphical presentation of the reported particle concentrations is given in Figure 3. With regard to accuracy, four results were questionable and three were unsatisfactory.

#### 2.1.3. Electron Microscopy, TEM and SEM

**Figure 4:**
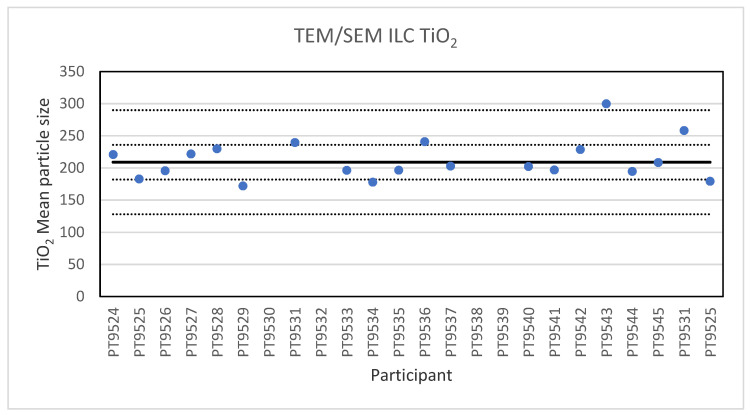
Results for the particle size of the TiO_2_ particle reported by the participants in the TEM/SEM ILC. The straight line indicates the consensus value, the dotted lines one and three times the robust standard deviation.

Electron microscopy is the only technique used in this series of ILCs with which NP can actually be directly imaged and which is capable of determining particle sizes as small as 1 nm. In this ILC, transmission as well as scanning electron microscopy (TEM/SEM) have been used. TEM is a high spatial resolution imaging and characterisation tool operating in a high vacuum. In a conventional TEM, a parallel beam of electrons is accelerated towards and transmitted through a thin sample (<100 nm). The electrons interact with the atomic structure of the sample which causes them to scatter, they exit the specimen and are projected onto a camera where an image is formed. TEMs are standard tools for measurements on the nanoscale for which standard procedures and reference materials exist [22]. An SEM scans a well-focused electron beam over a surface to produce magnified images of a specimen. The electrons in the beam interact with the sample, producing various signals that can be used to obtain information about the surface topography and composition. The electron beam probe sizes in SEM are in the range of 3–5 nm, which is not sufficient to resolve the smallest nanometre-scale details [23]. Tabletop SEM instruments are readily available, easy to use and their performance is getting steadily better. In the ILC for electron microscopy three particulate materials with different sizes were offered for analysis. These were 60 nm Au NPs in an aqueous suspension, 200 nm TiO_2_ particles and 40 nm BaSO_4_ NPs, both as solid powders. Twenty-two laboratories subscribed for participation in this ILC and twenty reported results. For the 200 nm TiO_2_ particles, reference material IRMM-388, the lowest reported value is 178 nm and the highest value is 300 nm. The consensus value is 209 nm while the robust standard deviation of the consensus value was 27 nm (RSD of 13%). This is slightly higher than the fit-for-purpose value of 10% of the consensus value (=21 nm). The uncertainty of the consensus value is 7.5 nm which exceeds 0.3σ_p_ and thus the uncertainty is taken into account, in the calculation of the z-scores. With regard to the accuracy, one of the results was questionable and one was unsatisfactory. Figure 4 presents the mean particle sizes reported by all participants.

**Figure 5:**
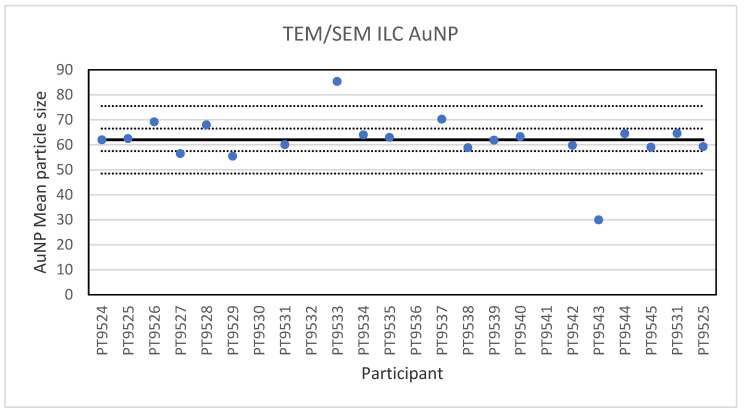
Results for the particle size of the AuNP particle reported by the participants in the TEM/SEM ILC. The straight line indicates the consensus value, the dotted lines one and three times the robust standard deviation.

For the 60 nm AuNPs, the lowest reported value was 30 nm and the highest value 85.4 nm. The consensus value is 62 nm with a robust standard deviation of 4.5 nm (RSD of 7.2%). The robust standard deviation is lower than the fit-for-purpose value of 10% of the consensus value (=6.2 nm). This is expected since this NM consists of spherical particles with a low tendency to agglomerate making sizing less operator dependent. The uncertainty of the consensus value is 1.3 nm which does not exceed 0.3σ_p_ and therefore the uncertainty is not taken into account in the calculation of the z-scores. With regard to the z-score, two results were unsatisfactory. One of these results was a reported particle size of 30 nm which is unlikely to be found with TEM and may be the result of a wrong calibration of the magnification. Figure 5 presents the results of all participants.

**Figure 6:**
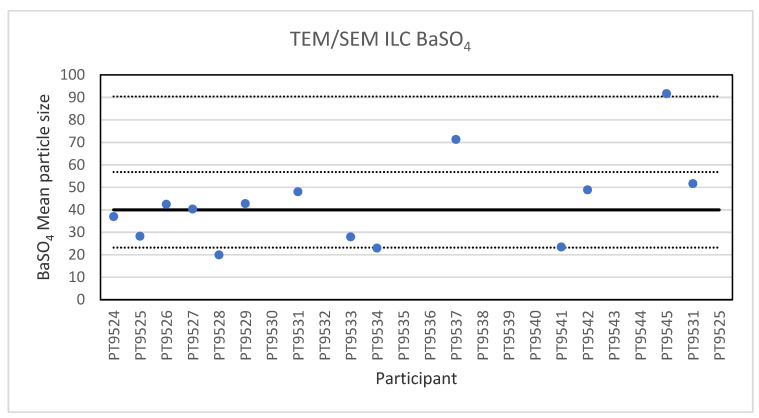
Results for the particle size of the BaSO_4_ particle NPs reported by the participants in the TEM/SEM ILC. The straight line indicates the consensus value, the dotted lines one and three times the robust standard deviation.

For the analysis of the 40 nm BaSO_4_ NPs, reference material IRMM-387, only fourteen laboratories reported results. This is probably a sign of the high degree of challenge in the measurement task. These particles, which are more or less spherical, are not only smaller but they also show a strong tendency to agglomerate, which makes the determination of the individual particle size challenging. The lowest reported particle size was 20 nm and the highest 91.7 nm. The consensus value is 40 nm with a robust standard deviation of 16.8 nm (RSD of 42%). This is in excellent agreement with the specified size of the reference material which is reported as 40.4 ± 20.2 nm. The robust standard deviation is twice the fit-for-purpose value of 20% of the consensus value (=8.0 nm). The uncertainty of the consensus value is 5.6 nm which exceeds 0.3σ_p_ thus the uncertainty is taken into account in the calculation of the z-scores. With regard to the accuracy, one of the results was questionable and two were unsatisfactory. Figure 6 presents the results of all participants.

#### 2.1.4. Other Techniques, DLS and AF4

**Figure 7:**
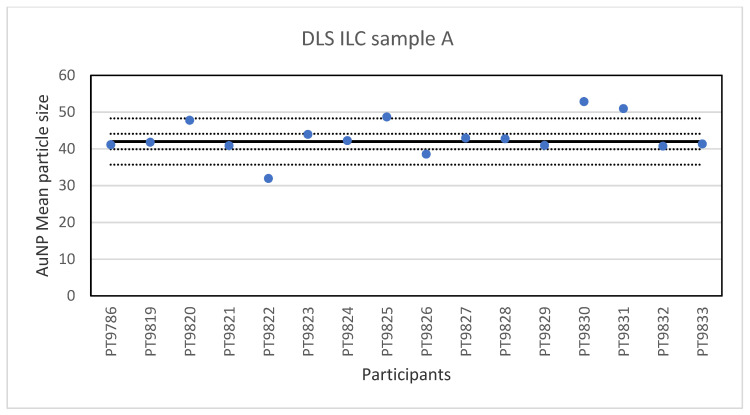
Results for the particle size in sample A reported by participants in the DLS ILC. The straight line indicates the consensus value, the dotted lines one and three times the robust standard deviation.

While PTA, spICP-MS and TEM/SEM were the only technique capable of reporting a number-based particle size distribution, two more ILCs were organised utilising other much used techniques to determine particle size, DLS and AF4. DLS is an ensemble technique for measuring the size of particles that are typically sub-micron and dispersed in a liquid. The particle size is determined by measuring the translational diffusion coefficient of the particles in the liquid which is the velocity of Brownian motion. The diffusion coefficient is then used to calculate the hydrodynamic diameter, which is the diameter of the particles including the solvent molecules which move with the particles [24]. In this ILC the participants were asked to determine the particle size in three different samples of particles: sample A containing a single-sized spherical gold particle with a size <50 nm; Sample B containing a single-sized spherical gold particle >50 nm; Sample C containing a mixture of the two single-sized spherical gold particles. Sixteen laboratories reported results for the particle diameter in sample A. The lowest value was 32 nm and the highest value 52.9 nm. The results of all participants for sample A are shown in Figure 7.

**Figure 8:**
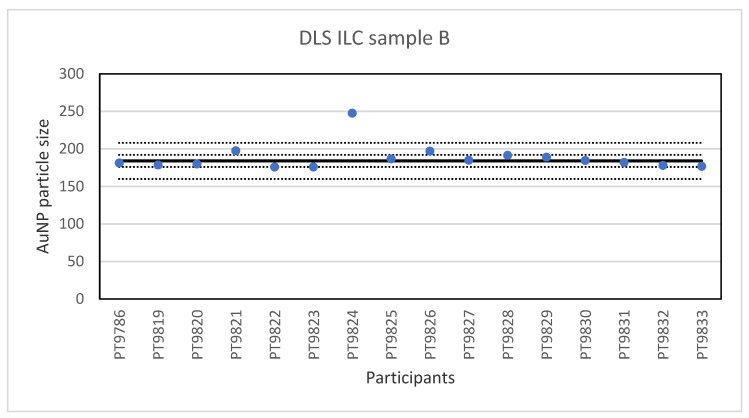
Results for the particle size in sample B reported by participants in the DLS ILC. The straight line indicates the consensus value, the dotted lines one and three times the robust standard deviation.

The consensus value is 42 nm with a robust standard deviation of 2.1 nm. This is exactly half the fit-for-purpose value of 10% of the consensus value (=4.2 nm). The uncertainty of the consensus value is 0.65 nm which does not exceed 0.3σp and therefore the uncertainty is not taken into account in the calculation of the z-scores. With regard to the accuracy, three results were questionable. The DLS measurement results for sample B are presented in Figure 8. For sample B, the lowest value reported was 176 nm and the highest value 247.7 nm. The consensus value is 184 nm with a robust standard deviation of 8.0 nm. As for sample A, this is less than half the fit-for-purpose value of 10% of the consensus value (=18 nm). The uncertainty of the consensus value is 2.5 nm which does not exceed 0.3σ_p_. With regard to accuracy, one result was unsatisfactory.

To make the determination more challenging the participants were also asked to measure particle sizes in a 1:1 mixture of the particle populations from samples A and B. In this case, fifteen laboratories reported results. For the smaller particle fraction, participants reported 14.3 nm as the lowest value and 54.3 nm as the highest value. The consensus value is 42 nm (equal to the single-sized particle measurement) with a robust standard deviation of 7.9 nm. This is almost twice the fit-for-purpose value of 10% of the consensus value (=4.2 nm). In addition, the uncertainty of the consensus value was 2.5 nm which exceeds 0.3σ_p_, thus the uncertainty is taken into account in the calculation of the z-scores. With regard to the accuracy, one result was questionable and one result was unsatisfactory. For the larger particle population, the lowest particle size value reported was 8.61 nm and the highest value 268.7 nm. The consensus value is 176 nm (almost equal to the single-sized particle measurement) with a robust standard deviation of 29 nm. This is more than 1.5 times higher than the fit-for-purpose value of 10% of the consensus value (=18 nm). The uncertainty of the consensus value is 9.3 nm which exceeds 0.3σp, thus the uncertainty is taken into account in calculating the z-scores. With regard to the accuracy, two results were questionable and one result was unsatisfactory.

**Figure 9:**
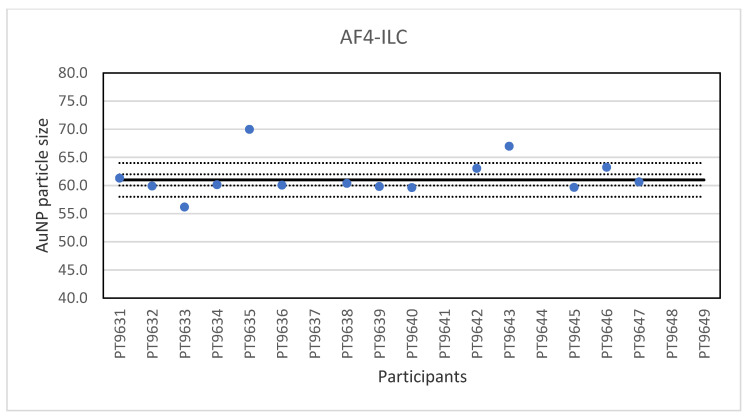
Results for the particle size reported by participants in the AF4 ILC. The straight line indicates the consensus value, the dotted lines one and three times the robust standard deviation.

Another ILC was organised for AF4 which is a fractionation method used for the characterisation of NP, polymers and proteins [25]. It is a separation technique based on a cross-flow perpendicular to the carrier liquid which exits at a constant rate through a semi-permeable wall on the bottom of a channel. As particles flow along the channel, the cross-flow separation field pushes the molecules towards the bottom of the channel. At the same time, the particles undergo a counter-acting diffusion back into the channel and the extent to which the molecules can diffuse back into the channel is dictated by their natural Brownian motion, a characteristic based on particle size. Smaller particles have a higher Brownian motion than larger ones and are able to diffuse higher into the channel and therefore elute earlier than larger particles. In this way, the retention time is related to the particle size. General guidelines on AF4 application have recently been published by ISO [26]. Samples were prepared from NanoComposix 60 nm gold NP by dilution in the same citrate matrix as the original material, and then bottled in vials. The vials for the participants were randomly selected and coded through a website application. The remaining vials were used for stability testing and were stored in the refrigerator. A set of suspensions of citrate-stabilised gold nanoparticles with particle sizes from 20 to 100 nm produced from BBI materials was included to prepare a particle size calibration curve. Participants were encouraged to use their in-house method and practical experience to select the most appropriate instrument settings. Nineteen laboratories registered for participation and fourteen reported results for the particle size. The lowest value reported was 55.6 nm and the highest value 70 nm. The consensus value is 61 nm with a robust standard deviation of 1.0 nm. This is six times lower than the fit-for-purpose value of 10% of the consensus value (=6.1 nm). The uncertainty of the consensus value is 0.34 nm which does not exceed 0.3σ_p_ and thus uncertainty is not taken into account in the calculation of the z-scores. With regard to the z-score accuracy, one result was questionable. The results of the particle size of the gold NP in water are shown in Figure 9. Eleven of the fourteen labs that reported results showed optimal performance by reporting particle sizes with satisfactory z-scores.

### 2.2. Measurements in Consumer Products

The first consumer product that was selected for this ILC was a sunscreen lotion which, according to the declaration on the packaging, contained nano-sized TiO_2_ particles. The second consumer product was a toothpaste which, according to the declaration on the packaging, contained TiO_2_ without mentioning whether this was nano-sized or not.

Thirty-two participants registered for this ILC and 26 reported results. Measurement techniques used were PTA, spICP-MS and TEM/SEM. Eight labs applied more than one measurement technique. A target standard deviation for proficiency test assessment (σ_p_) for the mean particle size and D_50_ value was set at 20%.

Eight laboratories reported results for the mean particle size of TiO_2_ particles in the sunscreen as determined with PTA. The reported particle sizes varied from 97 nm to the highest value of 251 nm. It should be noted that 251 nm was an outlier from one laboratory and that 97 to 141 nm was the most occurring reported size range across the participating laboratories. The consensus value is 124 nm with a robust standard deviation of 26 nm (RSD of 21%). This is comparable to the fit-for-purpose value of 20% of the consensus value (=25 nm). The uncertainty of the consensus value is 11.4 nm which exceeds 0.3σ_p_, thus the uncertainty is taken into account when calculating the z-scores. With regard to the accuracy, seven of the laboratories scored good results with satisfactory z-scores between −1 and +1. Not surprisingly, the results for the D_50_ values were comparable. The lowest reported value was 88 nm and the highest value 244 nm. The consensus value is 120 nm with a robust standard deviation of 26 nm (RSD of 22%). Again, this is comparable to the fit-for-purpose value of 20% of the consensus value (=24 nm). The uncertainty of the consensus value is 11.7 nm which exceeds 0.3σ_p_ and thus the uncertainty is taken into account. Regarding accuracy, seven laboratories scored satisfactorily.

**Figure 10:**
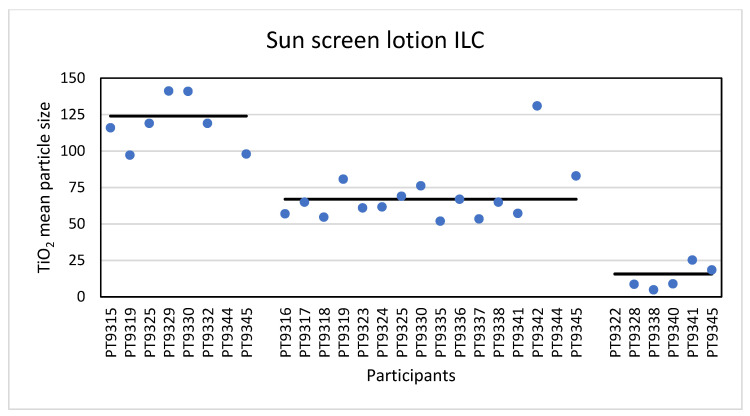
Results for the mean particle size reported by participants in the sun screen lotion sample. From left to right the results for PTA, spICP-MS and TEM/SEM are shown. The straight lines indicate the consensus values.

The results for the mean particle size of the participants (except the outlier of 251 nm) are shown in Figure 10.

Sixteen laboratories reported results for mean particle size and D_50_ determined with spICP-MS. For the mean TiO_2_ particle size the lowest reported value was 52 nm and the highest 227 nm. The consensus value is 67 nm with a robust standard deviation of 13.2 nm (RSD of 20%). This is comparable to the fit-for-purpose value of 20% of the consensus value (=13.4 nm). The uncertainty of the consensus value is 4.1 nm which only just exceeds 0.3σ_p_, thus the uncertainty is taken into account. With regard to the accuracy, fourteen laboratories scored good results with satisfactory z-scores in the range of −1 to +1. Only two laboratories reported unsatisfactory results with z-scores > 3. Thirteen of the sixteen laboratories that reported results for spICP-MS reported D_50_ values. The lowest value reported was 34 nm while the highest reported value was 228 nm, leading to a consensus value of 59 nm with a robust standard deviation of 16 nm (RSD of 27%). This is higher than the fit-for-purpose value of 20% of the consensus value (=11.0 nm). The uncertainty of the consensus value is 5.5 nm which exceeds 0.3σ_p_, thus the uncertainty is taken into account in the statistical evaluation. With regard to the accuracy, the results of two of the thirteen laboratories were unsatisfactory while for the other eleven laboratories satisfactory z-scores between −2 and +1 were calculated. According to the D_50_ value of 59 nm the conclusion is that the TiO_2_ particles in the sunscreen lotion are an NM and this was reported by eleven of the thirteen laboratories. The results for the mean particle size of all participants (except the outlier of 227 nm) are shown in Figure 10.

Finally, the samples were analysed using TEM/SEM by six of the laboratories. The lowest value reported was 4.9 nm and the highest value 251 nm. The consensus value is 15.7 nm with a robust standard deviation of 11.3 nm (RSD of 72%). This is more than 3.5 times higher than the fit-for-purpose value of 20% of the consensus value (=3.1 nm). The uncertainty of the consensus value is 5.8 nm which exceeds 0.7σ_p_. When the uncertainty is >0.7σ_p_, statistical evaluation is not possible. For D_50_ the lowest reported value was 4.5 nm while the highest value was 196 nm. The consensus value is 14.0 nm with a robust standard deviation of 9.8 nm (RSD of 70%) and an uncertainty value of 5.0 nm. As for the data of the mean particle size, statistical evaluation is not possible. From the D_50_ value it is concluded that the TiO_2_ material in the sunscreen sample is a NM and five of the six laboratories reported D_50_ values < 100 nm confirming this. The results for the mean particle size of all participants (except the outlier of 251 nm) are shown in Figure 10.

**Figure 11:**
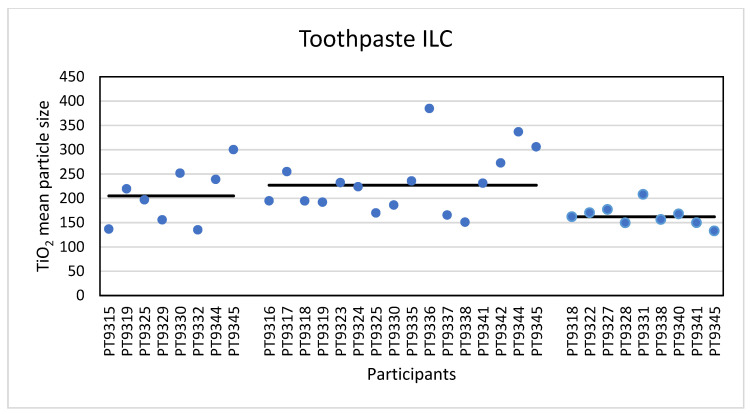
Results for the mean particle size reported by participants in the toothpaste sample. From left to right the results for PTA, spICP-MS and TEM/SEM are shown. The straight lines indicate the consensus values.

The second consumer product that was analysed in this ILC was a toothpaste product containing TiO_2_ particles. Eight laboratories reported results for the analysis of this material with PTA. An example of the toothpaste size distribution by PTA is shown in the Supplementary Information. For the mean particle size the lowest reported value was 135 nm and the highest value was 300 nm. The consensus value is 205 nm with a robust standard deviation of 69 nm (RSD of 34%). This is more than 1.5 times the fit-for-purpose value of 20% of the consensus value (=41 nm). The uncertainty of the consensus value is 31 nm which exceeds 0.7σ_p_ and as a consequence statistical evaluation is not possible. For the D_50_ value the lowest value was 130 nm and the highest value 358 nm. The consensus value is 215 nm with a robust standard deviation of 55 nm (RSD of 26%). This is only slightly higher than the fit-for-purpose value of 20% of the consensus value (=43 nm). The uncertainty of the consensus value is 24 nm which exceeds 0.3σ_p_ and therefore the uncertainty was taken into account in the statistical evaluation. With regard to the accuracy, seven of the eight results were satisfactory and one was questionable. According to the D_50_ value the particles in the toothpaste are not an NM and indeed all laboratories reported a D_50_ value > 100 nm. The results for the mean particle size of all participants are shown in Figure 11.

For the determination of the mean particle size with spICP-MS, sixteen laboratories reported results. The lowest value reported was 151 nm and the highest value 385 nm. The consensus value is 227 nm with a robust standard deviation of 60 nm (RSD of 26%). This is slightly higher than the fit-for-purpose value of 20% of the consensus value (=45 nm). The uncertainty of the consensus value is 18.7 nm which exceeds 0.3σ_p_, thus the uncertainty is taken into account. With regard to the accuracy, fourteen results were satisfactory, one was questionable and one result was unsatisfactory. Thirteen laboratories reported results of the D_50_ value using spICP-MS. The lowest value reported was 137 nm and the highest value was 365 nm. The consensus value is 252 nm with a robust standard deviation of 58 nm (RSD of 23%). This is comparable to the fit-for-purpose value of 20% of the consensus value (=50 nm). The uncertainty of the consensus value is 20 nm which exceeds 0.3σ_p_, thus the uncertainty is taken into account. With regard to the accuracy, two results were questionable. According to the consensus value of 252 nm the particles in the toothpaste are not an NM and, consistent with that, all laboratories reported D_50_ values > 100 nm. The results for the mean particle size of all participants are shown in Figure 11.

In total, nine laboratories reported results for TEM/SEM. The lowest reported mean particle size was 133 nm and the highest value 208 nm with a consensus value of 162 nm and with a robust standard deviation of 18.2 nm (RSD of 11%). This is almost half the fit for purpose value of 20% of the consensus value (=32 nm). The uncertainty of the consensus value is only 7.6 nm which does not exceed 0.3σ_p_ and therefore uncertainty is not taken into account. With regard to accuracy, all results were satisfactory with z-scores between −1 and +2. The lowest reported value for D_50_ was 125 nm and the highest was 195 nm. The consensus value is 150 nm with a robust standard deviation of 15.8 nm (RSD of 11%). As with the mean particle size, this is almost half the fit-for-purpose value of 20% of the consensus value (= 30 nm). The uncertainty of the consensus value is only 6.6 nm and since this does not exceed 0.3σ_p_, the uncertainty is not taken into account in the statistical evaluation. As before, with regard to accuracy, all results are satisfactory. According to the D_50_ value, this material is not an NM and all laboratories reported D_50_ values > 100 nm. The results for the mean particle size of all participants are shown in Figure 11.

## 3. Discussion

In the field of nanotechnology, analytical characterisation plays a vital role in understanding the behaviour and toxicity of NMs. Characterisation needs to be thorough and the technique chosen should be well-suited for the property to be determined, the material being analysed and the medium in which it is present. One way to test the performance of methods and laboratories is the organization of ILCs. The objective of the ACEnano project is not only to develop new methods and bring existing methods to a higher level of readiness, but also to test and benchmark existing methods for their performance. This is important since these methods are currently used to determine, for instance, whether a material is an NM or not, which under EU legislation leads to differences in terms of data needs for regulatory approval. In ACEnano the performance of these methods and laboratories are tested, starting with the analysis of pristine materials and/or aqueous suspensions of pristine materials, one ILC for each method. Following that, more complex samples, i.e., consumer products, were offered for analyses in a final ILC.

The first ILC was organised for the determination of particle size of Au NPs with PTA. With a lowest reported particle size of 58 nm and the highest value of 65 nm, the results of the ILC were very good. This was also the case for the robust standard deviation which was only 4% and much better than the fit-for-purpose value of 10%. The results are far better than in an earlier test round where it was found that the repeatability of multiple measurements on the instruments was generally good but the reproducibility was not, often resulting in deviating particle sizes. After the investigation, it was found that the systems reporting the largest size variations were either not maintained as recommended or the analysis was affected by inconsistency in sample preparation whereby the dilution step can create variations caused by different pipetting equipment, user operation and/or measurement set up including the flow cell not being clean, the wrong camera level being used, the image not being focused properly, and setting the analysis detection threshold incorrectly. As a follow-up it was decided to replace the older instruments by modern equivalents and to write an SOP for the measurements. In part, the good performance in this ILC will be the result of the fact that all participants were equipped with modern PTA instruments of the same type and configuration and a detailed SOP was provided to ensure consistency in sample preparation and analysis approach.

The second method selected for an ILC was spICP-MS. The results of the ILC look very promising, twenty-four out of twenty-six laboratories scored satisfactory results with particle sizes in the range of 52 to 74 nm. The two not satisfactory results reported particle sizes of 49.9 and 80 nm. Additionally, the robust standard deviation of 8% is better than the fit-for-purpose value of 10% and better than the reproducibility standard deviations of 20–35% that were achieved in an earlier ILC on the determination of silver NPs [13]. The results are comparable to the results obtained in an intercomparison for DLS and centrifugal liquid sedimentation (CLS) where between-laboratory standard deviations of 5–6% were achieved and in which the participants were carefully selected for their experience with DLS [27], while the participants in this ILC were not and did not receive any advice or training in spICP-MS. Therefore, we conclude that the results are satisfactory and that the laboratories that participated in the ILC are able to accurately determine the particle size of NP with spICP-MS. However, the determination of the particle number concentration produced far more deviating results than the mean particle diameter resulting in a robust standard deviation of 42%. The reason for this higher variation is not clear. One contributing factor could be that some participants reported particle numbers as determined (i.e., in the diluted preparation), some in the preparation and some in the original vial. To exclude this effect, some participants were asked to confirm the basis of their calculation and re-evaluate their results. A second contributing factor could be loss of particles over time, however, this was not indicated by the stability study which showed a small increase in the particle number concentration over time. A third contributing factor could be differences in the quality of the sample preparation (sonication) and dilution step. The relative robust standard deviation of 42% in this study is better than the between-laboratory standard deviation of 66–106% in an earlier ILC with Ag NPs in water [13]. While the results indicate that this determination is still difficult, it did show some improvement in time as labs became more experienced in using the method, and as hardware and software improved.

Electron microscopy is the only technique used in this series of ILCs with which NP can actually be directly imaged and which is capable of determining particle sizes as small as 1 nm. In this ILC, transmission as well as scanning electron microscopy (TEM/SEM) have been used. In general, the ILC for electron microscopy showed good results, wherein over 80% of the laboratories reported satisfactory results. In the cases where particles were spherical and showed a low tendency to agglomerate, such as the 60 nm Au NPs, accurate and precise results were produced. The particle sizes reported generally ranged from 55 to 70 nm with two outliers with sizes of 30 and 85.4 nm. The consensus value was 62 nm which compares well with the particle size provided by the supplier which is 60 ± 6 nm. In the case of irregular shaped particles, such as the 200 nm TiO_2_ particles, the robust standard deviation was higher than the fit-for-purpose value of 10%. One reason for this is probably that the irregular shape and a tendency to form agglomerates makes sizing of individual particles much more operator-dependent. Nevertheless, the consensus value of 209 nm was very close to the specified particle size of the reference material of 215.7 ± 56.3 nm. Difficulties were also observed for the strongly agglomerating 40 nm BaSO_4_ particles where a robust standard deviation of 42% was found. As with the TiO_2_ particles, the consensus value of 40 nm was in excellent agreement with the specified size of the reference material of 40.4 ± 20.2 nm. In general, when particles had irregular shapes and/or showed a tendency to agglomerate it is clear that the particle size determination was more problematic, resulting in an increased variation in the reported particle size.

While PTA, spICP-MS and TEM/SEM were the only techniques capable of reporting a number-based particle size distribution, two more ILCs were organised utilising other popular techniques to determine particle size, DLS and AF4. The results for the single-size samples A and B show that DLS is very well capable of accurately determining the particle size of relatively monodisperse spherical particles. This is not surprising since long-established and dedicated instruments and ISO standards are available for this method. On average, the relative robust standard deviation was 4–5% which compares well with the results of an earlier intercomparison organised for DLS and in which between-laboratory standard deviations of 5–6% were achieved [27]. To make the determination more challenging, the participants were also asked to measure particle sizes in a 1:1 mixture (sample C) of the particle populations from samples A and B. While the particle sizes found for the small and large particle in sample C were almost the same as in the single-size samples A and B, the variation in the results was much larger. The relative robust standard deviation was 16–19% compared to the 4–5% in the single-size measurements. The results clearly indicate that measuring particle size in the presence of different sized particles is more challenging and leads to higher relative robust standard deviations, in this case about four times larger compared to the single-sized particle measurements.

Another ILC was organised for AF4 which is a fractionation method used for the characterisation of NP, polymers and proteins. Samples were prepared from 60 nm Au NP by dilution in the same matrix as the original material. A set of suspensions of citrate-stabilised gold NP with particle sizes from 20–100 nm were included to prepare a particle size calibration curve. Participants were encouraged to use their in-house method and practical experience to select the most appropriate instrument settings. With particle sizes reported ranging from 55.6 to 70 nm and a very low relative robust standard deviation of about 2% the results of the ILC were excellent.

It is interesting to compare the results of PTA, spICP-MS, TEM/SEM and AF4 for a well-defined spherical particle such as the 60 nm Au particle since these four methods use different principles and do not determine the same kind of particle diameter. PTA and AF4 determine the hydrodynamic diameter of the particle while spICP-MS determines a mass-based spherical equivalent diameter and TEM/SEM a geometrical diameter [28]. The results of the independent ILCs were as follows: PTA 62 ± 2.3 nm; spICP-MS 61 ± 4.9 nm; TEM/SEM 62 ± 4.5 nm; AF4 61 ± 1.0 nm. As expected, these results are in good agreement, even using these different methods and agree well with the manufacturer’s specification of 60 ± 6 nm.

Finally, two consumer products, a sun screen lotion and a toothpaste, were selected for an ILC allowing the participants to use PTA, spICP-MS, TEM/SEM or a multiple of these techniques to determine the particle size of the TiO_2_ particles in these products. Participants were asked to determine the mean particle size and the D_50_ value, the median particle size where 50% of the particles is smaller than that size and 50% larger. This, of course, is related to the EU definition of an NM for regulatory purposes where, for 50% or more of the particles in the number size distribution, one or more external dimensions are in the size range of 1–100 nm. As a consequence, if the D_50_ value is below 100 nm the material is an NM and this should have been declared on the packaging of the product.

**Figure 12:**
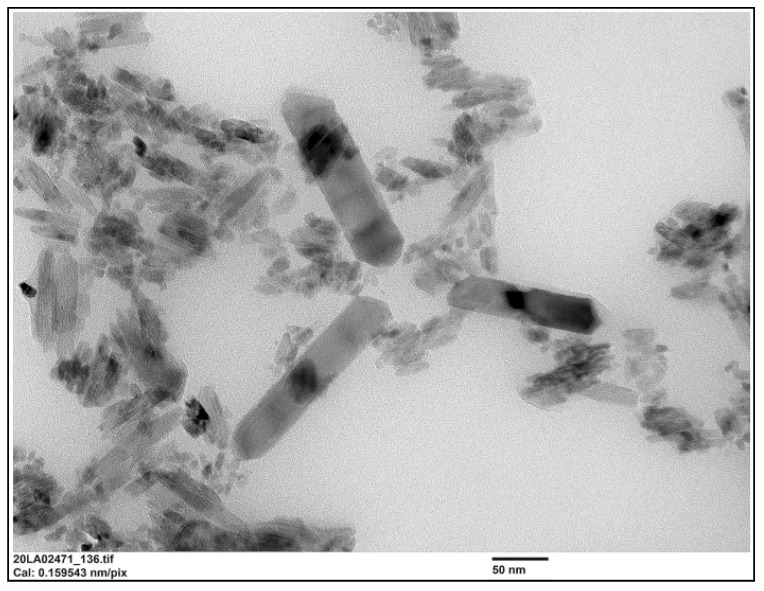
TEM image of the small elongated TiO_2_ particles in the sunscreen lotion sample. The larger particles in the centre of the image are ZnO particles (according to EDX analysis).

Eight laboratories reported results for the TiO_2_ particles in the sunscreen product as determined with PTA. The consensus value for mean particle size was 124 nm with a robust standard deviation of 26 nm. This is comparable to the fit-for-purpose value. The consensus value for D_50_ was 120 nm and six of the participating laboratories scored a D_50_ > 100 nm. From the D_50_ value as determined with PTA, it would be concluded that the particles in the sunscreen lotion are not an NM. However, it was found out later that the sunscreen lotion contained not only TiO_2_ particles but also ZnO particles which were not declared on the packaging. While the particle number concentration of the ZnO particles was much lower than that of the TiO_2_ particles, the particle sizes of the ZnO particles were much larger than the TiO_2_ particles as shown in the TEM image in Figure 12.

Since the PTA analysis is not particle composition-specific, the presence of the larger ZnO particles may have influenced the results for mean particle size and D_50_, biasing both to larger particle sizes. On the other hand, the larger variation may be due to using mean size results only. The mean particle size is commonly not the most suitable measure for PTA characterisation. As PTA is a particle-by-particle and relatively low number sampling technique, the mean size is very sensitive to sampling changes and often too variable to give robust characterisation results. It is always recommended to refer to the size distribution for sample complexity and polydispersity information. An example of the sunscreen lotion particle size distribution by PTA is shown in the Supplementary Information. Two of the eight laboratories reported D_50_ values that were below 100 nm and would have concluded that the particulate material in the sunscreen lotion is an NM. Since the particle concentration of the ZnO particles in the sunscreen lotion was very low, it may be that in these two cases the aliquot used for the instrumental analysis did not contain these ZnO particles.

Sixteen laboratories reported results for the mean particle size while thirteen reported results for D_50_ determined with spICP-MS. Unlike PTA, the determination with spICP-MS is composition-specific and spICP-MS specifically determines the size of the TiO_2_ particles, even in the presence of ZnO particles. According to the D_50_ value of 59 nm the conclusion is that the TiO_2_ particles in the sunscreen lotion are an NM and this was reported by eleven of the thirteen laboratories.

Finally, the samples were analysed using TEM/SEM by six of the laboratories. Since most of the laboratories used EDX to confirm the identity of the particles, they were able to specifically determine the mean particle size of the TiO_2_ particles. The consensus value is 15.7 nm with a robust standard deviation of 11.3 nm. This is more than 3.5 times higher than the fit-for-purpose value of 20% of the consensus value. The high robust standard deviation is a result of one significantly deviating result of 251 nm for particle size. Probably this laboratory did not size the much smaller TiO2 particles but the much larger ZnO particles, without probing them with EDX. If this result is removed from the dataset, a consensus value of 13.3 nm and a robust standard deviation of 4.2 nm is achieved, which is still higher than the fit-for-purpose value of 20%. This indicates that even with TEM/SEM it is still difficult to size such small particles, which may in part be a result of the fact that the TiO_2_ particles in the sample were not spherical but had an elongated shape. This can be seen in Figure 12 which shows a TEM image of the TiO_2_ particles in the sunscreen lotion. In this picture are also visible three much larger ZnO particles, which have a similar aspect ratio to the TiO_2_ particles. For D_50_, the consensus value is 14.0 nm with a robust standard deviation of 9.8 nm. From the D_50_ value it is concluded that the TiO_2_ material in the sunscreen sample is a NM and five of the six laboratories that reported D_50_ values < 100 nm confirm this.

The use of different measurement techniques leads to different mean particle sizes and D_50_ values. In the sunscreen lotion the consensus value for the mean particle sizes ranges from 15.7 nm for TEM/SEM to 67 nm for spICP-MS and 124 nm for PTA. For the D_50_ these consensus values range from 14.0 nm for EM to 59 nm for spICP-MS and 120 nm for PTA. This is expected since the particles in the samples consist of constituent (or primary) particles, aggregates (chemically bound constituent particles) and agglomerates (physically bound constituent particles and aggregates). The presence of such aggregates and agglomerates in the sunscreen material is clear from the TEM image in Figure 12. Using TEM/SEM, the constituent particles can be observed reasonably, even in aggregates and agglomerates, and as a consequence the true small constituent particle sizes in the sunscreen sample are reported by TEM/SEM. In spICP-MS, constituent particles and aggregates cannot be differentiated while agglomerates probably break down to constituent particles and aggregates in the measurement process. As a result, larger particle sizes are found by spICP-MS which resemble the size of aggregates in the material. For PTA this seems to be more pronounced. One reason for this may be that the small TiO_2_ particles as observed with TEM/SEM are below the detection limit of the PTA instrument. In addition, PTA determines the hydrodynamic particle diameter which is always larger compared to the mass-based diameter determined by spICP-MS and the geometrical diameter determined by TEM/SEM, interpreted by many EM users as the Minimum Feret diameter [29]. Note in Figure 12 that all particles are more or less rod-shaped and such asymmetry makes these particles challenging for tracking, however PTA will still report the sphere equivalent hydrodynamic diameter. It is reasonable to conclude that the presence of the larger rod-shaped ZnO particles in the sample have contributed to the larger average particle diameters determined by PTA.

**Figure 13:**
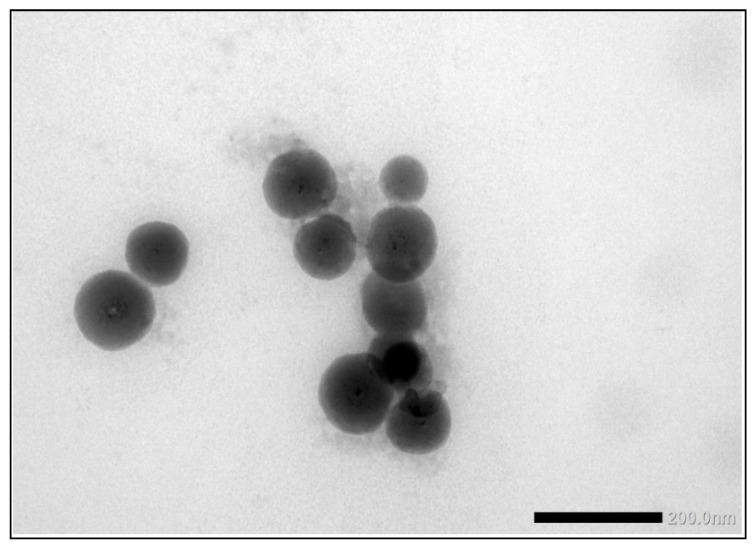
TEM image of the TiO_2_ particles in the toothpaste sample. While these are more discrete particles than in the sunscreen lotion, some aggregation is observed.

The second consumer product that was analysed in this ILC was a toothpaste product which, according to the declaration on the packaging contained TiO2 particles without mentioning whether this was nano-sized or not. Unlike the sunscreen lotion, the results of the mean particle sizes and D50 values for toothpaste are much closer together. For the mean particle size, the consensus values reported varied from 162 nm for TEM/SEM to 205 nm for PTA and 227 nm for spICP-MS. For the D50 values this was 150 nm for TEM/SEM to 215 nm for PTA and 252 nm for spICP-MS. While the results for PTA and spICP-MS are more or less comparable (and within the fit-for-purpose value of 20%) the results for EM are significantly smaller. This is probably again a result of the fact that only TEM/SEM can reasonably determine the true size of the constituent particles (even in the presence of aggregates and agglomerates). Moreover, as for the sunscreen lotion, EM users might have chosen the Minimum Feret diameter as the descriptor to be reported [29]. In the PTA and spICP-MS analysis constituent particles and aggregates cannot be differentiated and as a result higher particle sizes are found. Figure 13 shows a TEM image of the TiO_2_ particles as they were found in the toothpaste sample.

## 4. Materials and Methods

### 4.1. Study Concept

The aim of this study was to evaluate and benchmark the performance of the various methodologies in determining the size (and concentration where possible) of NMs. For that aim, laboratories around the world were invited to apply PTA, spICP-MS, TEM/SEM, DLS and AF4 methods. In the case of the spICP-MS technique, the precision of the particle number concentration was also evaluated. Between July 2018 and May 2020, six ILCs were organised. In detail, the PTA exercise was performed between April and July 2018, the spICP-MS in May to August 2018, the DLS in December 2018 to February 2019, the AF4 in March to May 2019, the TEM/SEM in June to September 2019, and a final ILC, in which participants were allowed to use PTA, spICP-MS and TEM/SEM, was organised in October 2019/May 2020. In general, participants were allowed to use their own standard (in house) procedures although reference was made to ISO and CEN documents in the instructions on sample handling and analysis that were sent together with the distributed test samples. No hands-on training was organised before the start of the ILCs. An exception is the PTA study for which the participants received state-of-the-art equipment of the same type and configuration, for which an operator training was organised, and a standard operating procedure (SOP) was provided along with all required materials.

### 4.2. Participants

This study describes the results of six independent ILCs performed by laboratories that are partners in the ACEnano project and extended with laboratories outside the ACEnano project. Invitations to participate in the individual ILCs were sent to the ACEnano partners, the Wageningen Food Safety Research (WFSR) nanotechnology network and the members of the WFSR proficiency testing database (ca. 2400 members). Some of the laboratories participated in more than one ILC. Each of the participants obtained a unique number for the performed ILC and all results were reported through the Laboratory Quality Services website of WFSR. An exception was the PTA study for which results were sent directly to the organiser, Malvern Panalytical. In detail, seven laboratories participated in the ILC analysing gold NP in water using the PTA technique. Six laboratories were ACEnano partners from Europe and one was from Asia. In the ILC analysing gold NP in water by spICP-MS, twenty-six laboratories participated and reported results. Among the participating laboratories, twenty-one were situated within Europe, three in Asia and two in North-America. In the ILC analysing gold NP in water by DLS, seventeen laboratories participated, of which thirteen were situated in Europe, three in Asia and one in North-America. In the ILC analysing gold NP in water by AF4, nineteen laboratories participated, from which seventeen were located in Europe, one in Asia and one in North-America. In the ILC involving the TEM/SEM techniques with gold, titanium dioxide and barium sulphate NP, twenty laboratories participated. Of these, fifteen laboratories were located in Europe, four in Asia and one in North-America. Finally, thirty-two participants subscribed to the ILC on the determination of TiO_2_ NP in consumer products using PTA, spICP-MS and TEM/SEM. Participants were able to report results for each applied technique. Of the thirty-two laboratories, twenty-six are situated within Europe and six in Asia.

### 4.3. Test Samples

The test samples used in this study consisted of gold nanoparticles (AuNPs), titanium dioxide particles (TiO_2_) and barium sulphate (BaSO_4_) NPs. In addition to these, a commercially available sunscreen lotion and toothpaste were used. In the performed proficiency tests, the AuNPs were purchased from two sources NanoComposix (San Diego, CA, USA) and BBI (Crumlin, UK). The TiO_2_ and BaSO_4_ materials were reference materials produced by JRC (Geel, Belgium) as part of the EU project NanoDefine and were kindly donated. The latter two were only used in the ILC for TEM/SEM. A summary of the properties of the tested materials is shown in Table 1.

In general, the AuNP materials were aliquoted (PTA ILC) or diluted, subsampled in glass vials (plastic vials for the PTA ILC), coded and shipped to the participants. The homogeneity of the diluted commercial AuNPs was tested according to the International Harmonized Protocol for Proficiency Testing of Analytical Laboratories [30] and ISO 13528 [31]. Ten randomly chosen samples were analysed in duplicate to determine the homogeneity of the particle concentration and particle size. It was demonstrated that the materials were sufficiently homogeneous for both tested parameters. The homogeneity of the TiO_2_ and BaSO_4_ reference materials was not tested since these were produced as reference materials by JRC, and, as such, had already been subjected to such analysis. The homogeneity of the consumer products was also not tested but the material was homogenized prior to subsampling for shipment to the participants. The stability of the aliquoted (PTA ILC) and diluted commercial AuNPs materials was determined by measuring the size of the materials at the beginning and the end of the individual ILCs. The stability of the TiO_2_ and BaSO_4_ materials was not determined as the samples were measured in a short period after dispersion by the laboratories. The stability of the consumer products was not measured because they were well within the product expiration date.

### 4.4. Sample and Measurement Protocol

For the PTA ILC the stock solutions of AuNPs were aliquoted into plastic vials and labelled prior to shipping. Along with the samples, the participants received all necessary consumables and reagents including a vial of filtered deionized water for further dilution of the samples. Malvern Panalytical coordinated the PTA ILC by materials distribution and results collection. Sample preparation and analysis steps were detailed in a SOP including dilution and data capture setup. It was advised to dilute the sample in water provided by Malvern Panalytical filtered prior to analysis, to provide the same sample environment and analysis conditions. The participants were requested to provide data and report the mode of the particle size as determined with the PTA instrument.

For the spICP-MS ILC the participants were informed that the material provided was a spherical gold NP with a particle size <100 nm and a mass concentration around 50 mg/L. Together with the samples, the participants received 1 g of citrate tribasic dihydrate (Sigma Aldrich, The Netherlands) to reconstitute the citrate buffer that the NanoComposix AuNPs are suspended in. The participants were asked to store the material in the refrigerator and allow it to come to room temperature 60 min prior to analysis. It was advised to dilute the material in the 1 mM citrate buffer but no dilution factor was indicated. The participants were requested to report the mean particle size as determined by spICP-MS. Participants of the TEM/SEM ILC received a 100 mg/mL stock solution of the NanoComposix AuNPs. JRC kindly donated the TiO_2_ and BaSO_4_ test materials and, following the coding of the samples, these were shipped as powdered materials as received from JRC. Participants were requested to report the mean particle sizes as determined with TEM and/or SEM.

For the DLS ILC participants received three separate samples with 40 nm particles (Sample A), 200 nm particles (Sample B) and a 1:1 *v*/*v* mixture of the two particles resulting in a bimodal sample (Sample C). The protocol stated that the samples A and B contained single-sized spherical colloidal particles <50 nm and >50 nm, respectively, while the sample C contained a mixture of the two sized spherical gold colloids. The protocol stated that samples should be stored in the refrigerator and be allowed to warm to room temperature 60 min prior to analysis. Samples used for the DLS measurement should be sonicated and diluted 1:10 in deionized water. Participants were requested to report the particle sizes as determined with the DLS instrument. Participants of the AF4 ILC received a 50 mg/mL stock solution of the NanoComposix AuNPs. Additionally, the laboratories received citrate-stabilized AuNPs from BBI of sizes 20, 40, 80 and 100 nm, mass-based concentration 50 mg/mL each, to calibrate the AF4. Furthermore, 2 mL of Nova Chem surfactant 100 (Postnova Analytics GmbH, Landsberg am Lech, Germany) were distributed to the participants. No information was given for sample dilution. The participants were requested to report the mean particle size as determined with the AF4 instrument.

In the last ILC, two consumer products, a sunscreen lotion and a toothpaste were sent to the participants. The participants were informed that both materials contained TiO_2_ particles of unknown size and were requested to determine the particle size of the TiO_2_ particles using one or more of the PTA, spICP-MS, or TEM/SEM techniques. A sample preparation procedure was given for both matrices and was as follows: Toothpaste: Weigh out a subsample of 200 mg and mix with 20 mL of ultrapure water. Mix on a magnetic stirrer for 1 hr and subsequently sonicate in an ultrasonic bath for 15 min. Sunscreen lotion: Weight out a subsample of 200 mg and mix with 20 mL of ethanol. Vortex for 1 min and sonicate in an ultrasonic bath for 15 min. These suspensions may be used as such for the analysis using TEM/SEM. For analysis with PTA a further dilution of 500 times in ultrapure water was advised. For spICP-MS a further dilution of 10,000 times (toothpaste) and 100,000 times (sunscreen lotion) in ultrapure water was advised. In house methods should be used for the instrumental analyses using PTA, spICP-MS and TEM/SEM and references to CEN/TS 17273 and ISO/ TS 19590:2017 were included. The participants were requested to report the mean particle size and the D_50_ value (the median particle size where 50% of the particles is smaller than that size and 50% larger, a D_50_ value < 100 nm indicates a NM), both expressed in nm.

### 4.5. Statistical Evaluation

The statistical evaluation of the quantitative results was carried out according to the International Harmonized Protocol for the Proficiency Testing of Analytical Chemistry Participants [30], elaborated by ISO, IUPAC and AOAC and ISO 13528 [31] in combination with insights published by the Analytical Methods Committee regarding robust statistics [32,33]. For the evaluation of the quantitative results, the consensus value (x), the uncertainty of the consensus value (u), a standard deviation for proficiency assessment (σ_p_) and z-scores were calculated. The consensus value was determined using robust statistics [31,32,33]. The advantage of robust statistics is that all values are taken into account: outlying observations are retained, but given less weight. Furthermore, it is not expected to receive normally distributed data in a proficiency test. When using robust statistics, the data do not have to be normally distributed in contrast to conventional outlier elimination methods. The robust mean of the reported results of all participants, calculated from the iterative process that starts at the median of the reported results, using a cut-off value depending on the number of results, was used as the consensus value [32,33]. The uncertainty of the consensus value is calculated to determine the influence of this uncertainty on the evaluation of the participants. A high uncertainty of the consensus value will lead to a high uncertainty in the calculated participants’ z-scores. If the uncertainty of the consensus value and thus the uncertainty of the z-scores is high, the evaluation could indicate unsatisfactory method performance without any cause within the laboratory. In other words, wrong conclusions could be drawn regarding the performance of the participants from the calculated z-scores if the uncertainty of the consensus value is not taken into account. The uncertainty of the consensus value (the robust mean) is calculated from the standard deviation of the consensus value and the number of values for the calculation of the consensus value [31]. If the uncertainty of the consensus value is negligible (u < 0.3σ_p_) it is not included in the statistical evaluation, otherwise it is.

## 5. Conclusions

The results of a number of ILCs for the characterisation of NMs organised in the frame of the H2020 project ACEnano were presented and discussed. ILCs were organised for PTA, spICP-MS, TEM/SEM, DLS and AF4 for particles in simple medium and consumer products. The results of the analyses of pristine well-defined particles such as the 60 nm Au NPs showed that laboratories with different methods were well capable of determining reliably the sizes of the particles. Interestingly PTA, spICP-MS, TEM/SEM and AF4, while using different measuring principles, reported similar particle sizes for the 60 nm Au NP. When pristine but more complex particles were analysed (TEM/SEM) or when mixtures of particles were analysed (DLS) the results showed a larger variation and bias. As expected, the analysis of particles in consumer products resulted in larger variations in particle sizes within techniques and clear differences between techniques. From the results of the sunscreen lotion sample it is clear that only TEM/SEM was able to correctly detect and size the small particles with visual confirmation. spICP-MS and PTA were more sensitive for aggregates, while the mean results of PTA were influenced by the presence of other particles, in this case the larger ZnO particles. In general, spICP-MS and TEM/SEM were able to correctly identify the TiO_2_ particles in complex formulations such as toothpaste and sunscreen lotion as being representative of the EU regulatory definition of NMs. For the toothpaste samples, the results from the different techniques were closely aligned. While PTA and spICP-MS reported similar results, TEM/SEM reported a somewhat smaller particle size. Nevertheless, all three methods correctly identified the TiO_2_ particles in this material as non-nanoscale according to the EU regulatory definition of an NM.

In general, from the results of the organised ILCs we can conclude that analytical laboratories are well capable of determining particle sizes of NM, and that there are several techniques suitable to do this, albeit with individual strengths and limitations. Using techniques such as TEM/SEM and spICP-MS also enables determination of whether a particulate material in a consumer product is an NM or not for regulatory purposes. The more complex the sample the higher the need for multi-technology characterization in order to identify, confirm and classify a material as an NM. It should be noted that other methods, such as analytical centrifugation, which were not included in the present series of ILCs, can also offer reliable results for complex NM.

Finally, it should be highlighted that while the size measurement of monodisperse NM of regular, simple shape is straightforward for most methods, the analysis of NM of more complex shape, or particles ‘embedded’ as aggregates or agglomerates in more complex matrices as consumer products is challenging. Similarly, the determination of particle sizes in bimodal distributions of NMs remains challenging. For the latter categories of particles, not only suitable sample preparation procedures but also measurement and analysis procedures, documented into standard operating procedures, which are specific to the particular materials/products must be available. For many real-life particulate products such procedures must still be developed. The literature is still scarce with reliable, standardized procedures. Various joint activities in several research groups and international (pre-)standardisation bodies are in progress. The present paper tries to offer a realistic overview of the present possibilities and challenges in the particle size and particle concentration analysis for (nano)particles with different degrees of complexity when using different analysis methods.

## Figures and Tables

**Table 1:** A summary of the properties of the used materials in the different ILCs.

Material	Manufacturer	Used in ILC	Particle Size (nm)
60 nm AuNPs	NanoComposix	spICP-MS AF4 TEM/SEM	60 ± 6
60 nm AuNPs	BBI	PTA	60 ± 3
40 nm AuNPs	BBI	DLS	40 ± 3
200 nm AUNPs	BBI	DLS	200 ± 6
TiO_2_ (IRMM-388)	JRC	TEM/SEM	215.7 ± 56.3
BaSO_4_ (IRMM-387)	JRC	TEM/SEM	40.4 ± 20.2
Sunscreen lotion	Retail	PTA/spICP-MS/ TEM/SEM	TiO_2_ mentioned in ingredient list
Toothpaste	Retail	PTA/spICP-MS/ TEM/SEM	TiO_2_ mentioned in ingredient list
Additional particles used for the calibration of the AF4 channel:			
20 nm AuNP	BBI	AF4	21.9 ± 0.3
40 nm Au NP	BBI	AF4	40.6 ± 0.3
80 nm Au NP	BBI	AF4	77.7 ± 0.5
100 nm AuNP	BBI	AF4	104.6 ± 0.8

## Data Availability

All relevant data are included within the manuscript. The raw data are available as Supplementary Materials.

## Supplementary Materials

The following are available online, Word document, “Benchmarking the ACEnano toolbox for characterisation of nanoparticle size and concentration by interlaboratory comparison. Supplementary Information”.

## Sample Availability

No available.
