# Correction: Peters et al. Benchmarking the ACEnano Toolbox for Characterisation of Nanoparticle Size and Concentration by Interlaboratory Comparison. *Molecules* 2021, *26*, 5315

**DOI:** 10.3390/molecules27154849

**Published:** 2022-07-29

**Authors:** Ruud Peters, Ingrid Elbers, Anna Undas, Eelco Sijtsma, Sophie Briffa, Pauline Carnell-Morris, Agnieszka Siupa, Tae-Hyun Yoon, Loïc Burr, David Schmid, Jutta Tentschert, Yves Hachenberger, Harald Jungnickel, Andreas Luch, Florian Meier, Jovana Kocic, Jaeseok Kim, Byong Chon Park, Barry Hardy, Colin Johnston, Kerstin Jurkschat, Jörg Radnik, Vasile-Dan Hodoroaba, Iseult Lynch, Eugenia Valsami-Jones

**Affiliations:** 1 Wageningen Food Safety Research, Wageningen University & Research, Akkermaalsbos 2, 6708 WB Wageningen, The Netherlands; ingrid.elbers@wur.nl (I.E.); anna.undas@wur.nl (A.U.); eelco.sijtsma@wur.nl (E.S.); 2 School of Geography, Earth and Environmental Sciences, University of Birmingham, Edgbaston, Birmingham B15 2TT, UK; s.m.briffa@bham.ac.uk (S.B.); i.lynch@bham.ac.uk (I.L.); e.valsamijones@bham.ac.uk (E.V.-J.); 3 Malvern Panalytical, Enigma Business Park, Grovewood Road, Malvern, Worcestershire WR14 1XZ, UK; pauline.carnell-morris@malvern.com (P.C.-M.); agnieszka.siupa@malvern.com (A.S.); 4 Department of Chemistry, College of Natural Sciences, Hanyang University, Seoul 04763, Korea; taeyoon@hanyang.ac.kr; 5 Institute of Next Generation Material Design, Hanyang University, Seoul 04763, Korea; 6 CSEM, Centre Suisse d’Electronique et de Microtechnique SA, Bahnhofstrasse 1, 7302 Lanfquart, Switzerland; loic.burr@csem.ch (L.B.); david.schmid@csem.ch (D.S.); 7 German Federal Institute for Risk Assessment (BfR), Department of Chemical and Product Safety, Max-Dohrn-Strasse 8-10, 10589 Berlin, Germany; jutta.tentschert@bfr.bund.de (J.T.); yves.hachenberger@gmx.de (Y.H.); harald.jungnickel@bfr.bund.de (H.J.); andreas.luch@bfr.bund.de (A.L.); 8 Postnova Analytics GmbH, Rankine-Str. 1, 86899 Landsberg, Germany; florian.meier@postnova.com; 9 Department of Chemistry and Applied Biosciences ETH Zurich, Vladimir-Prelog-Weg 1, 8093 Zurich, Switzerland; jovanat@inorg.chem.ethz.ch; 10 Korea Research Institute of Standards and Science (KRISS), 267 Gajeong-ro, Yuseong-gu, Daejeon 34113, Korea; jaeseok.kim@kriss.re.kr (J.K.); bcpark@kriss.re.kr (B.C.P.); 11 Edelweiss Connect GmbH, Technology Park Basel, Hochbergerstrasse 60C, 4057 Basel, Switzerland; barry.hardy@edelweissconnect.com; 12 Department of Materials, University of Oxford, Begbroke Science Park, Begbroke Hill, Oxford OX5 1PF, UK; colin.johnston@materials.ox.ac.uk (C.J.); kerstin.jurkschat@materials.ox.ac.uk (K.J.); 13 Bundesanstalt für Materialforschung und-prüfung (BAM), Unter den Eichen 87, 12205 Berlin, Germany; joerg.radnik@bam.de (J.R.); dan.hodoroaba@bam.de (V.-D.H.)

Due to miscommunication a number of potential co-authors were not listed in the original publication [1]. The actual list of authors should be as follows:

**Original:**

Ruud Peters ^1,^*, Ingrid Elbers ^1^, Anna Undas ^1^, Eelco Sijtsma ^1^, Sophie Briffa ^2^, Pauline Carnell-Morris ^3^, Agnieszka Siupa ^3^, Tae-Hyun Yoon ^4,5^, Loïc Burr ^6^, David Schmid ^6^, Jutta Tentschert ^7^, Yves Hachenberger ^7^, Harald Jungnickel ^7^, Andreas Luch ^7^, Florian Meier ^8^, Jörg Radnik ^9^, Vasile-Dan Hodoroaba ^9^, Iseult Lynch ^2^ and Eugenia Valsami-Jones ^2^

^1^  Wageningen Food Safety Research, Wageningen University & Research, Akkermaalsbos 2, 6708 WB Wageningen, The Netherlands; ingrid.elbers@wur.nl (I.E.); anna.undas@wur.nl (A.U.); eelco.sijtsma@wur.nl (E.S.)
^2^  School of Geography, Earth and Environmental Sciences, University of Birmingham, Birmingham, Edgbaston B15 2TT, UK; S.M.Briffa@bham.ac.uk (S.B.); i.lynch@bham.ac.Uk (I.L.); E.ValsamiJones@bham.ac.uk (E.V.-J.)
^3^  Malvern Panalytical, Enigma Business Park, Grovewood Road, Malvern WR14 1XZ, UK; pauline.carnell-morris@malvern.com (P.C.-M.); agnieszka.siupa@malvern.com (A.S.)
^4^  Department of Chemistry, College of Natural Sciences, Hanyang University, Seoul 04763, Korea; taeyoon@hanyang.ac.kr
^5^  Institute of Next Generation Material Design, Hanyang University, Seoul 04763, Korea
^6^  CSEM, Centre Suisse d’Electronique et de Microtechnique SA, Bahnhofstrasse 1, 7302 Lanfquart, Switzerland; loic.burr@csem.ch (L.B.); david.schmid@csem.ch (D.S.)
^7^  Department of Chemical and Product Safety, German Federal Institute for Risk Assessment (BfR), Max-Dohrn-Strasse 8-10, 10589 Berlin, Germany; jutta.tentschert@bfr.bund.de (J.T.); yves.hachenberger@bfr.bund.de (Y.H.); harald.jungnickel@bfr.bund.de (H.J.); Andreas.Luch@bfr.bund.de (A.L.)
^8^  Postnova Analytics GmbH, Rankine-Str 1, 86899 Landsberg, Germany; florian.meier@postnova.com
^9^  Bundesanstalt für Materialforschung und -Prüfung (BAM), Unter den Eichen 87, 12205 Berlin, Germany; Joerg.radnik@bam.de (J.R.); Dan.Hodoroaba@bam.de (V.-D.H.)
* Correspondence: ruudj.peters@wur.nl

**This should be replaced with:**

Ruud Peters ^1,^*, Ingrid Elbers ^1^, Anna Undas ^1^, Eelco Sijtsma ^1^, Sophie Briffa ^2^, Pauline Carnell-Morris ^3^, Agnieszka Siupa ^3^, Tae-Hyun Yoon ^4,5^, Loïc Burr ^6^, David Schmid ^6^, Jutta Tentschert ^7^, Yves Hachenberger ^7^, Harald Jungnickel ^7^, Andreas Luch ^7^, Florian Meier ^8^, Jovana Kocic ^9^, Jaeseok Kim ^10^, Byong Chon Park ^10^, Barry Hardy ^11^, Colin Johnston ^12^, Kerstin Jurkschat ^12^, Jörg Radnik ^13^, Vasile-Dan Hodoroaba ^13^, Iseult Lynch ^2^ and Eugenia Valsami-Jones ^2^

^1^  Wageningen Food Safety Research, Wageningen University & Research, Akkermaalsbos 2, 6708 WB Wageningen, The Netherlands; ingrid.elbers@wur.nl (I.E.); anna.undas@wur.nl (A.U.); eelco.sijtsma@wur.nl (E.S.)
^2^  School of Geography, Earth and Environmental Sciences, University of Birmingham, Edgbaston, Birmingham B15 2TT, UK; s.m.Briffa@bham.ac.uk (S.B.); i.lynch@bham.ac.uk (I.L.); e.valsamiJones@bham.ac.uk (E.V.-J.)
^3^  Malvern Panalytical, Enigma Business Park, Grovewood Road, Malvern, Worcestershire WR14 1XZ, UK; pauline.carnell-morris@malvern.com (P.C.-M.); agnieszka.siupa@malvern.com (A.S.)
^4^  Department of Chemistry, College of Natural Sciences, Hanyang University, Seoul 04763, Korea; taeyoon@hanyang.ac.kr
^5^  Institute of Next Generation Material Design, Hanyang University, Seoul 04763, Korea
^6^  CSEM, Centre Suisse d’Electronique et de Microtechnique SA, Bahnhofstrasse 1, 7302 Lanfquart, Switzerland; loic.burr@csem.ch (L.B.); david.schmid@csem.ch (D.S.)
^7^  German Federal Institute for Risk Assessment (BfR), Department of Chemical and Product Safety, Max-Dohrn-Strasse 8-10, 10589 Berlin, Germany; jutta.tentschert@bfr.bund.de (J.T.); yves.hachenberger@gmx.de (Y.H.); harald.jungnickel@bfr.bund.de (H.J.); andreas.luch@bfr.bund.de (A.L.)
^8^  Postnova Analytics GmbH, Rankine-Str. 1, 86899 Landsberg, Germany; florian.meier@postnova.com
^9^  Department of Chemistry and Applied Biosciences ETH Zurich, Vladimir-Prelog-Weg 1, 8093 Zurich, Switzerland; jovanat@inorg.chem.ethz.ch
^10^  Korea Research Institute of Standards and Science (KRISS), 267 Gajeong-ro, Yuseong-gu, Daejeon 34113, Korea; jaeseok.kim@kriss.re.kr (J.K.); bcpark@kriss.re.kr (B.C.P.)
^11^  Edelweiss Connect GmbH, Technology Park Basel, Hochbergerstrasse 60C, 4057 Basel, Switzerland; barry.hardy@edelweissconnect.com
^12^  Department of Materials, University of Oxford, Begbroke Science Park, Begbroke Hill, Oxford OX5 1PF, UK; colin.johnston@materials.ox.ac.uk (C.J.); kerstin.jurkschat@materials.ox.ac.uk (K.J.)
^13^  Bundesanstalt für Materialforschung und-prüfung (BAM), Unter den Eichen 87, 12205 Berlin, Germany; joerg.radnik@bam.de (J.R.); dan.hodoroaba@bam.de (V.-D.H.)
* Correspondence: ruudj.peters@wur.nl

All co-authors agree with the content of this correction and wish to apologize for any inconvenience to the readers resulting from this error.
