# Supplementary material for: Benchmarking the ACEnano Toolbox for Characterisation of Nanoparticle Size and Concentration by Interlaboratory Comparisons
Source: Molecules. 2021 Sep 1;26(17):5315. doi: 10.3390/molecules26175315 (PMC8433974; doi:10.3390/molecules26175315)
Supplement: Supplementary file 1 [file molecules-26-05315-s001.zip › molecules-1291058-supplementary.pdf]

Benchmarking the ACEnano toolbox for characterisation of nanoparticle size and concentration by interlaboratory comparison. Supplemental info.

Ruud Peters[1], Ingrid Elbers[1], Anna Undas[1], Eelco Sijstma[1], Sophie Briffa[2], Pauline Carnell-Morris[3], Agnieszka Siupa[3], Tae-Hyun Yoon[4,5], Loïc Burr[6], David Schmid[6], Jutta Tentschert[7], Yves Hachenberger[7], Harald Jungnickel[7], Andreas Luch[7], Florian Meier[8], Jörg Radnik[9], Vasile-Dan Hodoroaba[9], Iseult Lynch[2], Eugenia Valsami-Jones[2].

[1] Wageningen Food Safety Research, Wageningen University & Research, Akkermaalsbos 2, 6708 WB Wageningen, The Netherlands
[2] School of Geography, Earth and Environmental Sciences, University of Birmingham, Birmingham, Edgbaston, B15 2TT, United Kingdom
[3] Malvern Panalytical, Enigma Business Park. Grovewood Road. Malvern. WR14 1XZ. United Kingdom.
[4] Department of Chemistry, College of Natural Sciences, Hanyang University, Seoul 04763, Republic of Korea
[5] Institute of Next Generation Material Design, Hanyang University, Seoul 04763, Republic of Korea
[6] CSEM, Centre Suisse d'Electronique et de Microtechnique SA, Bahnhofstrasse 1, 7302 Lanfquart, Switzerland
[7] German Federal Institute for Risk Assessment (BfR), Department of Chemical and Product Safety, Max-Dohrn-Strasse 8-10, 10589 Berlin, Germany
[8] Postnova Analytics GmbH, Rankine-Str 1, 86899 Landsberg, Germany
[9] Bundesanstalt für Materialforschung unf -prüfung (BAM), Unter den Eichen 87, 12205 Berlin, Germany

1. Interlaboratory comparisons test samples

The test samples used in this study consisted of gold nanoparticles (AuNPs), titanium dioxide particles (TiO_2) and barium sulphate nanoparticles ($BaSO_4$). In addition to these a commercially available sun screen lotion and toothpaste were used. In the performed proficiency tests the AuNPs were purchased from two sources NanoComposix (San Diego, CA, USA) and BBI (Crumlin, UK). The TiO_2 and $BaSO_4$ materials were reference materials produced by JRC (Geel, Belgium) as part of the EU project NanoDefine and kindly donated. The latter two were only used in the ILC for TEM/SEM. A summary of the properties of the tested materials is shown in Table S1.

Table S1. A summary of the properties of the used materials in the different ILCs.

Material	Manufacturer	Used in ILC	Particle diameter (nm)
60 nm AuNP	NanoComposix	spICP-MS<br>AF4<br>TEM/SEM	60 ± 6
60 nm AuNP	BBI	PTA	60 ± 3

40 nm AuNP	BBI	DLS	40 ± 3
200 nm AuNP	BBI	DLS	200 ± 6
TiO_2 (IRMM-388)	JRC	TEM/SEM	215.7 ± 56.3
$BaSO_4$ (IRMM-387)	JRC	TEM/SEM	40.4 ± 20.2
Sunscreen lotion	Shop	PTA/spICP-MS/TEM/SEM	TiO_2 mentioned in ingredient list
Toothpaste	Shop	PTA/spICP-MS/TEM/SEM	TiO_2 mentioned in ingredient list
Additional particles used for the calibration of the AF4 channel:			
20 nm AuNP	BBI	AF4	21.9 ± 0.3
40 nm AuNP	BBI	AF4	40.6 ± 0.3
80 nm AuNP	BBI	AF4	77.7 ± 0.5
100 nm AuNp	BBI	AF4	104.6 ± 0.8

2. Numerical results of the ILCs: Pristine materials

2.1 Measurements of pristine nanoparticles

At first pristine nanoparticles were used in the ILCs to determine whether methods and laboratories were able to determine accurate particle sizes in simple matrices before going to more complex matrices like consumer products. The techniques that were tested in these ILCs were PTA, spICP-MS, TEM/SEM, DLS and AF4. The numerical results are presented here.

2.2 Particle tracking analysis: 60 nm Au NP (BBI)

labcode	size
	nm
PT0001	62
PT0002	61
PT0003	63
PT0004	61
PT0005	58
PT0006	65
PT0007	64

2.3 Single particle ICP/MS: 60 nm Au NP (NanoComposix)

labcode	size
	nm
PT018	65.24

PT019	62.4
PT020	59.52
PT021	61
PT022	65
PT023	74
PT024	64
PT025	80
PT026	55.5
PT027	60
PT028	61.6
PT029	58.16
PT030	63
PT9892	68.8
PT9954	52
PT9955	57
PT9956	60.2
PT9957	61.6
PT9991	61.25
PT9992	66
PT9993	49.9
PT9994	63
PT9995	58.4
PT9996	54
PT9997	60
PT9998	66.4

labcode	particle concentration
	#/L
PT018	1.81815E+13
PT019	1.45E+13
PT020	1.503E+13
PT021	1.006E+13
PT022	1.57E+13
PT023	17300000
PT024	1.1E+13
PT025	7.61E+12
PT026	2.04E+13
PT027	1.9E+13
PT028	1.27E+13
PT029	1.6E+13
PT030	44300000

PT9892	1.01E+13
PT9954	2E+13
PT9955	4.1E+13
PT9956	1.34E+13
PT9957	1.7E+13
PT9991	1.61E+13
PT9992	1.135E+13
PT9993	detected
PT9994	6.73E+12
PT9995	2.4E+13
PT9996	2.1E+13
PT9997	1.69E+13
PT9998	7.98E+12

2.4 Electron microscopy, TEM and SEM: 60 nm Au NP (NanoComposix), IRMM-388, IRMM-387

labcode		size	size	size
		AuNP	TiO2	BaSO4
		nm	nm	nm
PT9524	TEM	62	221	37
PT9525	SEM	62.5	183	28.3
PT9526	SEM	69.2	195.9	42.5
PT9527	TEM	56.5	221.9	40.4
PT9528	TEM	68	230	20
PT9529	SEM	55.5	172.1	42.8
PT9530				
PT9531	TEM	60.1	239.7	48.1
PT9532				
PT9533	TEM	85.4	196.5	28
PT9534	TEM	64	178	23
PT9535		63	196.7	
PT9536			241.05	
PT9537	TEM	70.3	203	71.3
PT9538		58.85		
PT9539		61.89		
PT9540		63.3	202.4	
PT9541	TEM		197.1	23.5
PT9542	SEM	59.8	228.9	48.9
PT9543		30	300	
PT9544		64.5	194.7	
PT9545	TEM	59.1	208.6	91.7
PT9531	SEM	64.6	258.3	51.7

PT9525	TSEM	59.3	179.4	

2.5 DLS: 40 nm Au NP (sample A), 200 nm Au NP (sample B) and mixture (sample C) (BBI)

labcode	size	size	size	size
	batch A	batch B	small batch C	large batch C
	nm	nm	nm	nm
PT9786	41.19	181.3	47.6	200.6
PT9819	41.83	178.6	39.93	165.4
PT9820	47.84	180.02	42.43	158.61
PT9821	40.94	197.63	43.46	8.61
PT9822	32	176	35	176
PT9823	43.99	176	34.52	137.7
PT9824	42.3	247.7	54.3	268.7
PT9825	48.72	186.79	37.39	184.21
PT9826	38.6	197.1	21.4	162.8
PT9827	43	185	49	210
PT9828	42.77	191.5	47.9	198.7
PT9829	40.99	189	42.58	175.3
PT9830	52.9	184.5	14.3	99.6
PT9831	51	182		
PT9832	40.8	178	46.6	182
PT9833	41.4	176.8	47.6	193.1

2.6 AF4: 60 nm Au NP (NanoComposix)

labcode	size	size	size		size
	ms1	ms2	ms3		average
	nm	nm	nm		nm
PT9631	61.34				61.3
PT9632	59.6	59.8	60.4		59.9
PT9633	55.6	56.4	56.6		56.2
PT9634	59.7	60.4	60.4		60.2
PT9635	70	70	70		70.0
PT9636	60.2	60	60.1		60.1
PT9637	unable to report				
PT9638	60.14	60.69	60.41		60.4
PT9639	59.8481	59.8563	59.8741		59.9
PT9640	59.7	59.8	59.5		59.7
PT9641	unable to report				

PT9642	63.3	61.6	64.3		63.1
PT9643	64	74	63		67.0
PT9644	no response after receipt samples				
PT9645	60.33	59.34	59.41		59.7
PT9646	63	63.6	63.2		63.3
PT9647	57	63.8	61.2		60.7
PT9648	unable to report				
PT9649	unable to report				

3. Numerical results of the ILCs: Consumer products

3.1 Measurements in consumer products

The first consumer product that was selected for this ILC was a sunscreen lotion which, according to the declaration on the packaging, contained nano-sized titanium dioxide particles. The second consumer product was a toothpaste which, according to the declaration on the packaging contained titanium dioxide without mentioning whether this was nano-sized or not.

3.2 Numerical results of consumer products: Sunscreen lotion

labcode	sunscreen	sunscreen	sunscreen	sunscreen	sunscreen	sunscreen
	PTA	PTA	spICP-MS	spICP-MS	TEM/SEM	TEM/SEM
	mean	D50	mean	D50	mean	D50
	nm	nm	nm	nm	nm	nm
PT9315	116					
PT9316			57	49		
PT9317			65	57		
PT9318			54.7			
PT9319	97.21	88.4	80.8	72.7		
PT9322					251.2	195.5
PT9323			61	54.5		
PT9324			61.75	38.7		
PT9325	119	114	69			
PT9327						
PT9328					8.7	8.5
PT9329	141.2	136.6				
PT9330	140.99	134.87	76.21	45.37		
PT9331						
PT9332	119	114				
PT9335			52	46		
PT9336			67	60		
PT9337			53.5	61		
PT9338			65		4.9	4.5
PT9340					9	8

PT9341			57.3	50.7	25.3	25
PT9342			131	121		
PT9343		118				
PT9344	251	244	227	228		
PT9345	98	93.6	83	81	18.5	15.5

3.3 Numerical results of consumer products: Toothpaste

labcode	toothpaste	toothpaste	toothpaste	toothpaste	toothpaste	toothpaste
	PTA	PTA	spICP-MS	spICP-MS	TEM/SEM	TEM/SEM
	mean	D50	mean	D50	mean	D50
	nm	nm	nm	nm	nm	nm
PT9315	137					
PT9316			195	208		
PT9317			255	255		
PT9318			194.7		162.1	152.5
PT9319	219.7	207	192.2	214.6		
PT9322					170.5	153.8
PT9323			232.5	263.5		
PT9324			224	234		
PT9325	197	189	170			
PT9327					177.1	160.3
PT9328					149.6	137.9
PT9329	155.8	141.2				
PT9330	251.8	237.6	186.4	195.44		
PT9331					208.06	195.47
PT9332	135.4	130.3				
PT9335			236	247		
PT9336			385	365		
PT9337			165.8			
PT9338			151	137	156.5	143.6
PT9340					168	162
PT9341			231.4	257.7	149.9	135
PT9342			273	264		
PT9343		358				
PT9344	239	236	337	335		
PT9345	300.3	253.3	306	301	133	125

4. Particle size distributions by PTA: Consumer products

4.1 Particle size distribution by PTA of sunscreen lotion

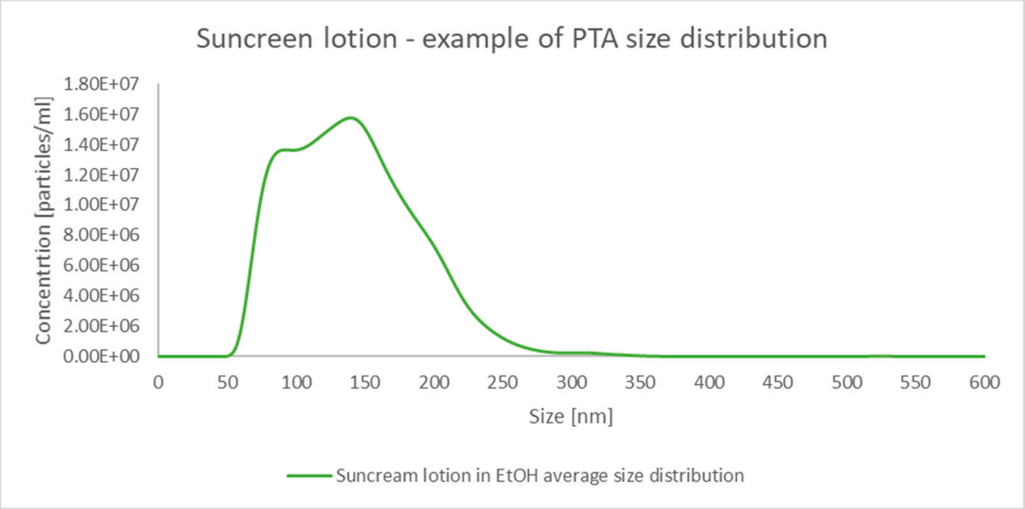

Example of the sunscreen lotion particle size distribution by PTA. PTA detected particles from 50 to 400 nm showing the sunscreen lotion to be a quite complex system of various size NPs. In this example the average reported mean size was 141 nm, average reported modal size was 116 nm while D_{50} was 137 nm.

4.2 Particle size distribution by PTA of toothpaste

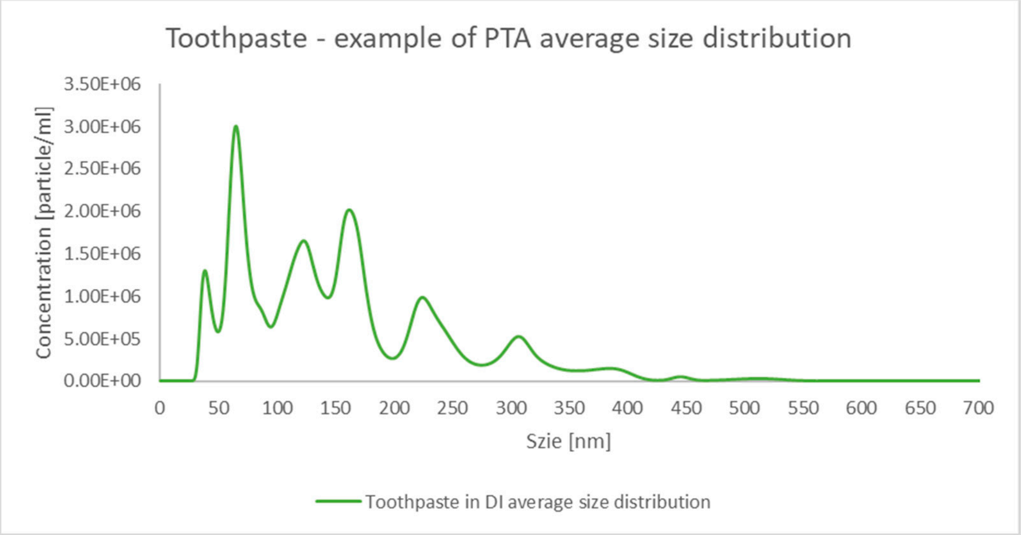

Example of the toothpaste particle size distribution by PTA. PTA detected particles from 30 to 550 nm showing the toothpaste to be quite a complex system of various size NPs. In this example the average reported mean size was 156 nm, average reported modal size was 92 nm while D_{50} was 141 nm.
